# New Neurons at Risk: Genotoxicants and Brain Development

**Published:** 2006-11

**Authors:** Ernie Hood

Neurodevelopmental disorders such as learning disabilities, mental retardation, and autism spectrum disorders affect an estimated 5–10% of the 4 million babies born in the United States annually. In a report released in 2000, the National Research Council concluded that 3% of these disorders are the direct result of environmental exposures to neurotoxicants, with another 25% arising from the interaction between such exposures and genetic susceptibility. Investigators have shown that many of these long-term adverse outcomes can be attributed to genetic damage to immature neurons in the developing brain related to exposure to genotoxicants, chemicals that disrupt the complex, delicate cellular process that regulates development of a fully functional brain. Although the precise mechanisms involved are still poorly understood, scientists are now starting to unravel the molecular ravages caused by genotoxicants **[*EHP* 114:1703–1712; Kisby et al.]**.

As reported in this month’s issue, a research group exposed cultures of immature neurons known as granule cells and the more developed and more abundant astrocytes to sublethal doses of two well-characterized alkylating genotoxicants: methylazoxymethanol (MAM), a highly toxic compound synthesized from the poison found in plants called cycads, and nitrogen mustard (HN2), a chemotherapeutic agent. The team then analyzed the cultures for cell viability, DNA damage, markers of apoptosis, and corresponding gene expression patterns. The intent of the research is ultimately to identify the key molecular networks that are targeted by genotoxicants, in order to understand how such agents influence brain development.

Results showed that granule cells were much more sensitive to the genotoxicants than astrocytes. The exposures caused dose-dependent DNA lesions that persisted and accumulated, apparently because, unlike astrocytes, granule cells lack the ability to repair DNA damage. In other words, once the exposure has wreaked havoc on the developing neurons, the damage is done, and its impact is felt throughout the process of neuronal development, leading to long-term impairment. The authors speculate that these events “could explain why the developing cerebellum is a prime target in several human neurodevelopmental disorders.”

The team also discovered that the two genotoxicants affected distinctly different sets and functional types of genes. MAM targeted differentiation, stress and immune response, cell signaling, and transcriptional regulation genes, whereas HN2 targeted apoptosis and protein synthesis gene expression. This preferential targeting suggests that different genotoxicants probably cause completely different effects in the developing brain. With a significant proportion of neurodevelopmental disabilities thought to stem directly from early exposures to DNA-damaging agents or gene–environment interactions related to such exposures, further investigation of the molecular networks involved in these effects is clearly needed.

## Figures and Tables

**Figure:**
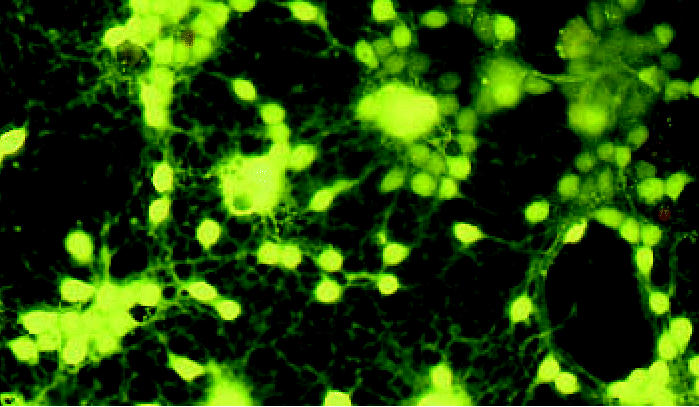
Young and vulnerable Immature neurons called granule cells are more sensitive to genotoxicants.

